# Efficacy and safety of tocilizumab in managing cytokine release syndrome after CD19 CAR-T therapy for relapsed or refractory B-cell acute lymphoblastic leukemia

**DOI:** 10.3389/fimmu.2025.1530623

**Published:** 2025-03-14

**Authors:** Qianyi Zhou, Yuxin An, Xiaomei Zhang, Xia Xiao, Xue Bai, Pengjiang Liu, Yedi Pu, Juanxia Meng, Haibo Zhu, Cuicui Lyu, Huan Zhang, Yu Zhang, Tianle Xie, Haotian Meng, Hairong Lyu

**Affiliations:** ^1^ First Center Clinical College, Tianjin Medical University, Tianjin, China; ^2^ Department of Hematology, Tianjin First Central Hospital, Tianjin, China; ^3^ Nankai University School of Medicine, Nankai University, Tianjin, China

**Keywords:** chimeric antigen receptor T (CAR-T) cell, tocilizumab, cytokine release syndrome (CRS), efficacy and safety, acute B-lymphoblastic leukemia (B-ALL)

## Abstract

**Purpose:**

CD19 chimeric antigen receptor T (CAR-T) cell therapy has shown promise in treating relapsed or refractory (R/R) B-cell acute lymphoblastic leukemia (B-ALL), but cytokine release syndrome (CRS) remains a significant side effect.

**Methods:**

This retrospective cohort study investigated the use of tocilizumab for managing CAR-T-related CRS in 45 R/R B-ALL patients.

**Results:**

Of these, 17 patients received tocilizumab, resulting in a significant reduction in the duration of grade 3 CRS compared to those who did not receive the drug. Additionally, 10 patients showed decreased cytokine levels.Importantly, tocilizumab did not impair CAR-T cell expansion or efficacy, nor did it increase the incidence of adverse events.

**Conclusion:**

These findings suggest that tocilizumab may be an effective and safe strategy for mitigating CAR-T-related CRS in R/R B-ALL patients, potentially improving patient outcomes and survival.

## Introduction

CAR-T cell therapy is an effective emerging therapy for treating hematological malignancies. Up till now, six CAR-T cell products have been approved by the Food and Drug Administration (FDA) (1–8). Among them, CAR-T cells targeting CD19 for B-cell malignancies has been widely and maturely applied in clinical practice, achieving a complete remission rate up to 90% (9, 10).

However, CAR-T cells can cause certain side effects, among which cytokine release syndrome (CRS) is the most common and serious (11). The symptoms of CRS include fever, hypoxia, hypotension, and so on (12). The American Society for Transplantation and Cellular Therapy (ASTCT) graded CRS based on symptoms (13). The mechanism of CRS is complex and still not fully understood, which may be related to the release of inflammatory factors by various immunocytes (11). The initial cytokines released lead to further cytokine release after activation of immune cells such as macrophages. This results in a cytokine storm. Of these, IL-6 is considered the most critical cytokine and the serum IL-6 levels may correlate with the severity of CRS (14). Due to the important role of IL-6, current guidelines recommend the use of tocilizumab (anti-IL-6R) for the prevention and treatment of CRS caused by CAR-T cells (15). Currently, there are more studies on the prevention and treatment of CRS with tocilizumab in lymphoma and myeloma patients (16–18), while in B-ALL patients relevant research is less. In addition, few studies have evaluated the side effects of tocilizumab in the treatment of CAR-T cell-induced CRS (19).

In summary, we enrolled 45 B-ALL patients who received CAR-T cell infusion and retrospectively studied the efficacy and safety of tocilizumab in the treatment of CRS through patients’ symptoms and serum biomarkers. We consider that tocilizumab does not affect the proliferation and efficacy of CAR-T cells, and there are no serious side effects.

## Materials and methods

### Patients and data inclusion

**Figure 1:**
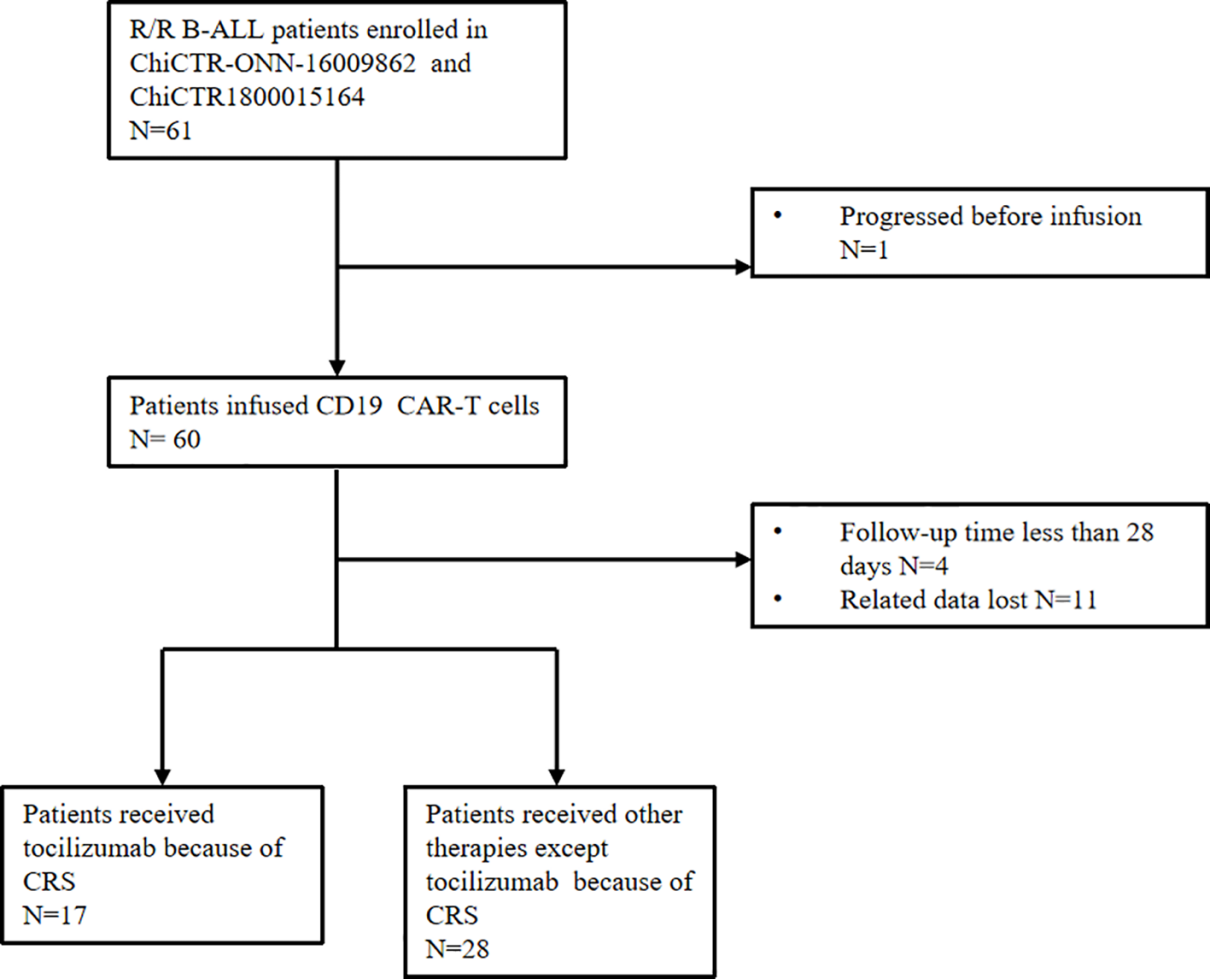
Inclusion criteria and study patient cohort.

According to the inclusion/exclusion criterion of two clinical trials (ChiCTR-ONN-16009862 and ChiCTR1800015164), we conducted a retrospective analysis of 61 eligible patients with R/R B-ALL from April 2020 and October 2023. The exclusion criteria for follow-up were as follows (1): patients progressed before infusion (2); the follow-up time less than 28 days (3); the related data lost. In the end, 16 patients were excluded and 45 patients were enrolled in this study, 17 patients of which were treated with tocilizumab infusion (**Figure 1**).

This study protocol was reviewed and approved by the Ethics Committee of Tianjin First Central Hospital and was registered for clinical trials in the China Clinical Trial Registration Center. All participations signed written informed consent before enrollment.

### Manufacturing and infusion of CD19 CAR-T cells

The peripheral blood mononuclear cells were obtained from patients or healthy donors. CD3+ T cells were isolated by magnetic beads and cultured them in culture medium which contains CD3/CD28 stimulating beads. After activation, we transduce lentiviral vector including CD19 CAR into these cells. The CD19 CAR contained a 4-1BB costimulatory domain and a CD3-ζ signaling domain constructed as previously described (20). Finally, CAR-T cells were expanded and evaluated for transduction efficiency (approximately 50%). Prior to infusion (Day 0), all patients received lymphodepleting chemotherapies. (300mg/m^2^/d cyclophosphamide and 30mg/m^2^/d fludarabine for 3 to 2 days) (21).

### Definitions and management of CRS

CRS is the main toxicity observed in B-ALL patients treated with CAR-T cells. We graded CRS according to the ASTCT consensus (the grading criteria is detailed in **Table 1**). We classified it into mild CRS (grade 1-2 CRS) and severe CRS (grade 3-4 CRS). In addition to symptomatic treatments such as oxygen therapy, antipyretics and vasopressors, tocilizumab and corticosteroid are common drugs used in the treatment of CRS. We regarded fever that could not be explained by other causes and the increasing of cytokines as the beginning of CRS. However, the optimal timing of tocilizumab is not yet clear, and it is most commonly recommended for patients with symptoms such as refractory or persistent hypotension, hypoxia, etc. (22) In our study, tocilizumab or corticosteroid is given to patients as determined by the physicians, within 12 hours of the appearance of symptoms.

**Table 1:** The ASTCT consensus of grading CRS.

Grade	Fever	Hypotension	Hypoxia
1	Temperature≥38°C	×	×
2	Temperature≥38°C	not requiring vasopressors	and/or requiring low-flow nasal cannula or blow-by
3	Temperature≥38°C	requiring one vasopressor with or without vasopressin	and/or requiring high-flow nasal cannula^, facemask, nonrebreather mask, or Venturi mask
4	Temperature≥38°C	requiring multiple vasopressors (excluding vasopressin)	and or requiring positive pressure (eg: CPAP, BiPAP, intubation and mechanical ventilation)

(#) Fever that could not be explained by other causes. And it is no longer necessary for subsequent CRS grading after treatment with tocilizumab or corticosteroid (^) Low-flow nasal cannula is 6 L/min and high-flow nasal cannula is > 6 L/min.

### Assessment of other related diseases

ICANS is the second observed side effect in R/R B-ALL patients treated with CD19 CAR-T. We recorded the different ICANS symptoms and the exact time of symptom appearance, then graded ICANS according to the ASTCT consensus. We also focused on the occurrence of hematological toxicity. In addition, impairment of liver and kidney function was also observed in a small percentage of patients, which were also recorded and evaluated. Finally, the infection of these patients was diagnosed based on clinical presentation and etiological examination.

### Assessment of response and survival

Response to CAR-T cells was assessed by bone marrow morphological analysis, flow cytometry, and genetic testing on day 28. Complete response (CR) was defined as <5% bone marrow blasts in morphology regardless of cell count recovery. And it was further classified into MRD+CR or MRD-CR based on minimal residual disease (MRD). The efficacy of tocilizumab or corticosteroid was assessed by serial monitoring of temperature, ferritin, C-reactive protein (CRP) and cytokines (including IL-2, IL-6, IL-10, TNF-α (tumor necrosis factor-α), etc). The end of CRS was considered to be the disappearance of fever and a decrease in cytokines. Finally, we evaluated the progression-free survival (PFS) and overall survival (OS) of these two groups of patients (toci group and non-toci group).

### Statistical analyses

The characteristics of patients were analyzed using descriptive statistics. All measurement data were described in terms of median and range and compared by using t tests or Mann-Whitney tests. Enumeration data were described as frequencies (%) and compared using chi-squared tests or Fisher’s exact tests. Statistical analyses were performed using the SPSS v26.0 software (Chicago, IL, USA) and the GraphPad Prism v9 software (GraphPad, La Jolla, CA, USA).

## Results

### Essential characteristics of patients

From April 2020 to October 2023, a total of 61 patients with R/R B-ALL were proposed to receive CAR-T cells. Among them,1 case experienced disease progression before receiving CAR-T therapy, eventually 60 patients received CAR-T therapy. Among these 60 cases, excluding 4 cases with follow-up time less than 28 days, and 11 cases with relevant data loss, finally 45 cases were included in our retrospective study. Baseline characteristics of all 45 patients and the subgroup analysis of 2 groups (toci group and non-toci group) were summarized (**Table 2**). Of the total 45 patients, 53.3% (24/45) were male, the median age was 36 years (range 8–67), and 84.8% (38/45) were high-risk phenotype or genotypes. The median number of therapy lines before CAR-T cell infusion was 2 (range, 1–3). As shown in the table, the two groups were very similar in terms of gender, genotypic risk level, previous treatments and dose of CAR-T cells. The differences were not statistically significant. However, the toci group had a higher tumor load, CRS grade and dose of corticosteroid with significant statistical difference. We also performed a logistic regression analysis of these potential factors (**Supplementary Table S4**). We found that none of the factors were statistically significant. In this study, there was insufficient evidence of an association between these factors and the use of tocilizumab in treatment.

**Table 2:** Baseline characteristics of all 45 patients and the subgroup analysis of 2 groups.

Characteristic	Total (N = 45)	Toci (N = 17)	Non-toci (N = 28)	P	P^#^
Median age (range), years	36 (8-67)	42 (14-67)	32 (8-61)	0.033	0.33
Male, No. (%)	24 (53.3)	8 (47.1)	16 (57.1)	0.511	5.11
High-risk phenotype or genotypes, No. (%)	38 (84.4)	14 (82.4)	24 (85.7)	1.000	10
Median lines of therapy (range)	2 (1-3)	2 (1-3)	2.5 (1.5-3.5)	0.395	3.95
Allogeneic SCT, No. (%)	9 (20.0)	3 (17.6)	6 (21.4)	1.000	10
Bone marrow blasts, No. (%)				0.042	0.42
<30	31 (68.9)	8 (47.1)	23 (82.1)		
30-50	5 (11.1)	3 (17.6)	2 (7.1)		
>50	9 (20.0)	6 (35.3)	3 (10.7)		
Median blasts (range)	24.0 (0-96.65)	34.15 (0-93.99)	17.78 (0-96.65)		
Doses of CAR-T (range) (×10^6^/kg)	2.1 (0.15-5.06)	1.5 (0.3-3.37)	2.38 (0.15-5.06)	0.106	1.06
Grade of CRS, No. (%)				0.019	0.19
0	4 (8.9)	0 (0)	4 (14.3)		
1	7 (15.6)	0 (0)	7 (25.0)		
2	19 (42.2)	8 (47.1)	11 (39.3)		
3-5	15 (33.3)	9 (52.9)	6 (21.4)		
ICANS, No. (%)	2 (4.4)	2 (11.8)	0 (0)	0.267	2.67
Dose of methylprednisolone-equivalent corticosteroid, mg, (range)	40 (0-800)	60 (30-201)	0 (0-80)	0.004	0.04

SCT, stem cell transplantation; #, Bonferroni-adjusted P-value.

### Efficacy of CAR-T cells and post-infusion management

**Figure 2:**
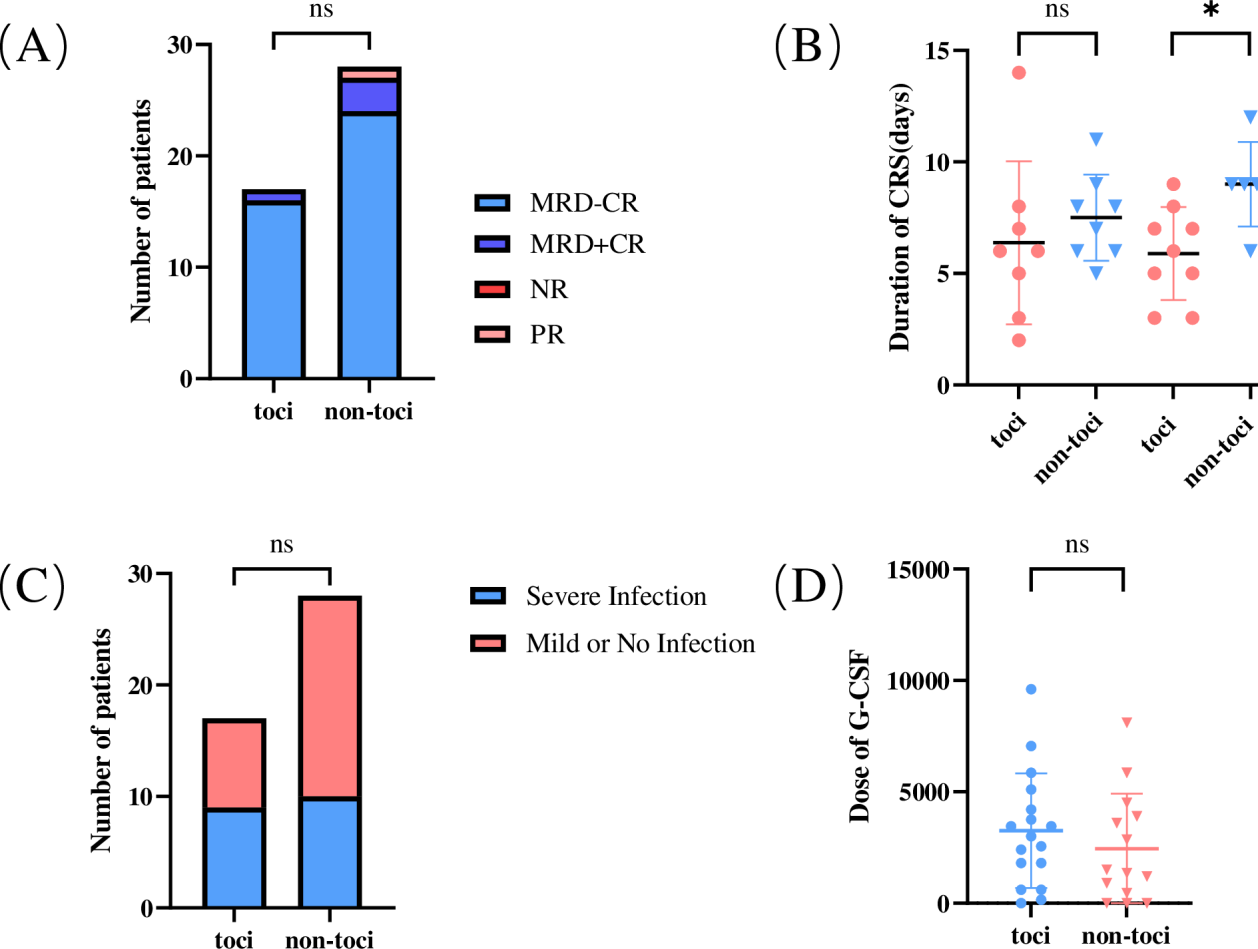
Efficacy and safety of using tocilizumab to treat CRS. **(A)** Comparison of efficacy between the two groups of patients at day 28 after receiving CD19 CAR-T therapy; **(B)** The duration of CRS between the two groups. We compared grade 1-2 CRS separately from grade 3 CRS; **(C)** Comparison of the incidence of serious infections in the two groups; **(D)** Comparison of the dose of G-CSF in the two groups, we regarded high doses of G-CSF as more severe hematologic toxicity. *p < 0.05. CR, complete response; MRD, minimal residual disease; PR, partial response; NR, non-remission. ns, no significance, P>0.05.

All 45 patients received CD19 CAR-T cells. Of the total 45 patients, 88.9% (N=40) achieved MRD-CR, 8.9% (N=4) achieved MRD+CR, and 2.2% (N=1) achieved partial remission (PR) (**Figure 2A**). After infusion, 91.1% (41/45) of patients had various grades of CRS, including 7 patients in grade 1, 19 patients in grade 2, and 15 patients in grade 3. There were no grade 4 CRS events. The median duration of CRS in patients in toci group was 6.1 (range, 2-14) days. In comparison, the median duration in non-toci group was 5.5 (range, 0-12) days. And ICANS occurred in two patients (4.5%). One patient developed grade 3 ICANS characterized by syncope and vision decrease that lasted 7 days.

Tocilizumab was applied to 17 patients, including 8 patients with grade 2 CRS and 9 patients with grade 3 CRS. The administration of tocilizumab was mostly within 12 hours after the onset of CRS-related symptoms, and a repeated dose of tocilizumab was given if the symptoms were not significantly relieved 8 hours after infusion. One to six doses of tocilizumab were used to suppress CRS with median dose of 480 (range, 160–800) mg. The dosing range for tocilizumab was 24-48 hours. Corticosteroids were used in 25 patients, and the cumulative methylprednisolone-equivalent corticosteroid dose was 100 mg (range, 7.5–302.5). A total of 10 patients in the non-toci group were treated with corticosteroid only, including 1 patient with grade 1 CRS, 3 patients with grade 2 CRS and 6 patients with grade 3 CRS. There were no cases of ICANS, nor any instances of grade 4 CRS or higher. In combination with the clinical symptoms, tocilizumab was not chosen due to the physician’s decision and the financial constraints of a part of the patients.

### Effectiveness of tocilizumab in treating CRS

Clinical symptoms of CRS in patients after CAR-T cells infusion include unexplained fever, hypotension and hypoxemia. These symptoms are the main basis for grading according to ASTCT criteria. Accordingly, the clinical presentation of the patients was documented and the duration of CRS was calculated, with particular attention to the toci group. For grade 3 CRS, we found that the duration in the toci group was significantly shorter than non-toci group, and the difference was statistically significant. But there was no significant difference in duration between the two groups in grade 1-2 CRS (**Figure 2B**).

**Figure 3:**
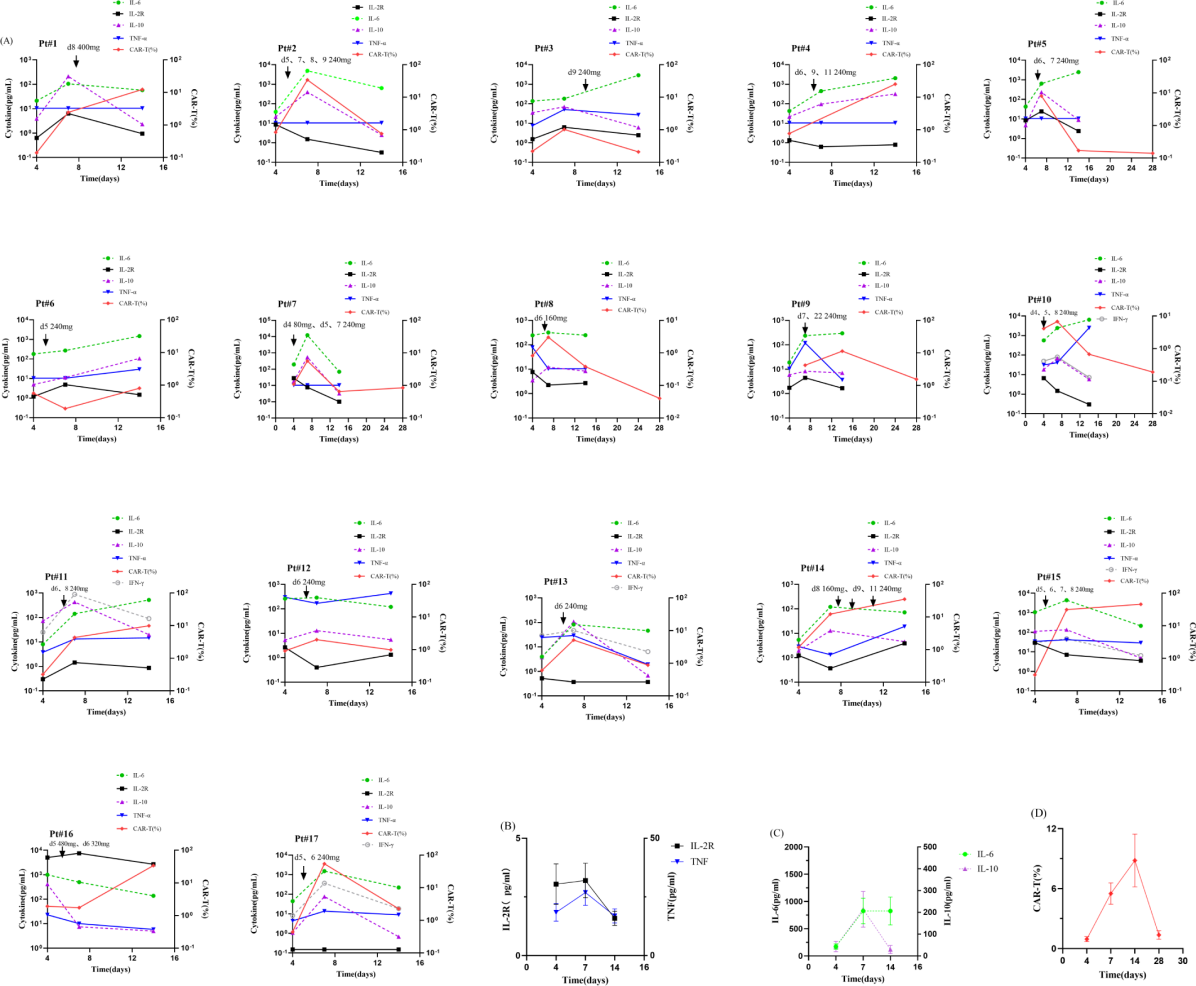
**(A)** Cytokines, CAR-T ratio of toci group patients. **(B-D)** The overall trends of Cytokines, CAR-T ratio in toci group. We consider the time of the first CAR-T cell infusion as day 0. The Cytokines included IL-2R, IL-6, IL-10 and TNF-α. A few patients also included data about IFN-γ.

The serum cytokine levels were correlated with CRS levels, so we continuously monitored changes of serum cytokine levels for 17 patients in the toci group. (**Figure 3**) 58.8% (10/17) patients exhibited a reduction in cytokine levels following tocilizumab treatment. Meanwhile, their clinical symptoms associated with CRS were markedly alleviated. We consider that this illustrates a substantial therapeutic role of tocilizumab in these patients.

Nevertheless, 41.2% (7/17) patients still showed elevated serum level of IL-6 after application of tocilizumab. Concurrently, their clinical manifestations showed no significant improvement. This may be the result of a number of factors. First, serum level of IL-6 is affected by CAR-T cell expansion. Pt 4 and Pt 11 applied tocilizumab at day 6、9、11 and day 6、8, respectively, while they had persistent expansion of CAR-T cell until day 15. Secondly, the acute graft-versus-host disease after infusion of donor-derived CAR-T cell also has some effects. Pt 5 developed acute intestinal GVHD and her symptoms resolved after treatment with ruxolitinib. Subsequently her serum level of IL-6 also exhibited a reduction. Finally, infection is also a very important influencing factor. Pt 3、6、9、10 showed different sites of infection, such as pneumonia, cholecystitis, etc. After adjustment for antibiotics and supportive care, Pt 、9、10 were stable after infection control, while Pt 6 died of respiratory failure due to severe lung infection.

Overall, the majority of 7 patients had a transient and mild increase in serum levels of IL-6. No patients developed ICANS due to elevated serum IL-6 levels. And it is not clear how tocilizumab affects the levels of other cytokines.

### Safety of tocilizumab in treating CRS

**Figure 4:**
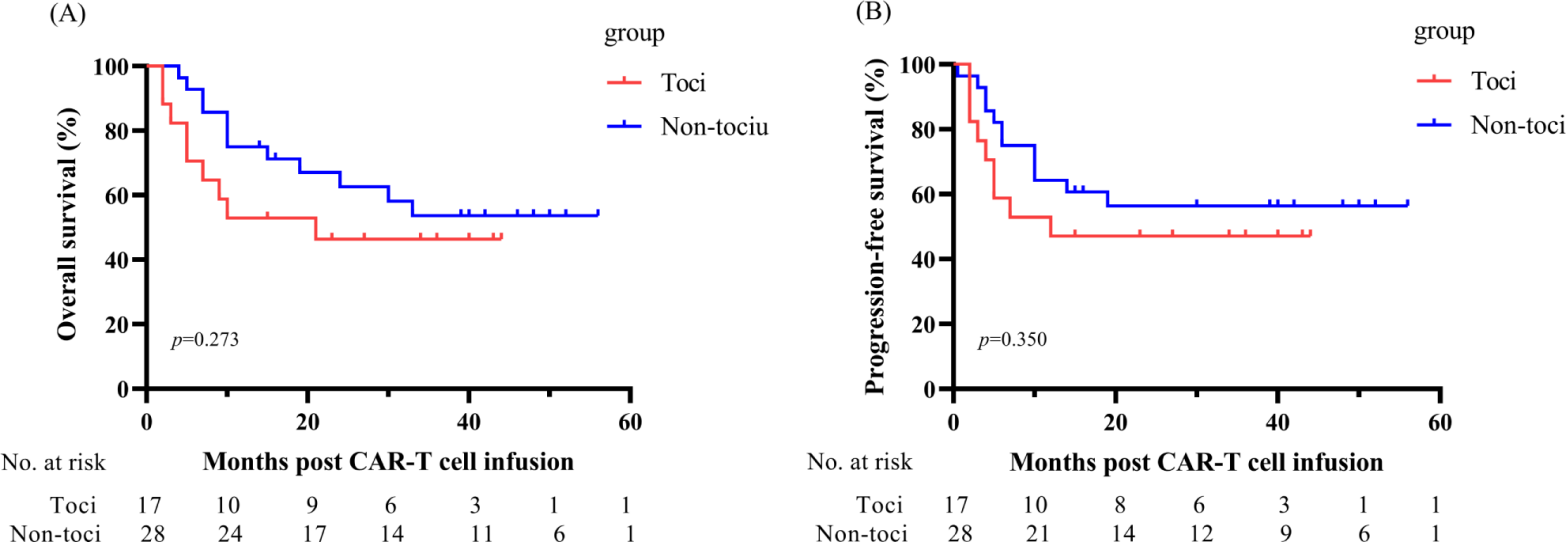
Relationship between receipt of tocilizumab with OS **(A)** and PFS **(B)** in patients with acute B-cell lymphoblastic leukemia after CD19 CAR-T therapy. OS, overall survival; PFS, progression-free survival.

Although tocilizumab is effective in relieving symptoms of severe CRS, the effect of tocilizumab on CAR-T cell expansion and efficacy is controversial. In our study, 94.12%(n=16)of the toci group and 85.71%(n=24)of the non toci group obtained MRD-CR. There was no statistically significant difference in the CR rate between toci and non-toci groups (p=0.616). In addition, there was no statistical difference in PFS (p=0.350) and OS (p=0.273) between the two groups (**Figure 4**). Meanwhile, tocilizumab didn’t affect the expansion of CAR-T cells (**Supplementary Figure S1**).

Moreover, tocilizumab does not increase the incidence of side effects. There were 52.9%(n=9)of patients in toci group and 35.7%(n=10)of patients in non-toci group developed serious infections. The differences were not statistically significant (p=0.353) (**Figure 2C**). In terms of hematological toxicity, we also compared the dosage of granulocyte colony stimulating factor (G-CSF) used between two groups to further verify the safety of tocilizumab, but the results still showed no statistically significant difference (p=0.227) (**Figure 2D**). In the **Supplementary Materials**, we also demonstrated that tocilizumab has no significant effect on liver and kidney function (**Supplementary Figure S2**).

## Discussion

CD19 CAR-T therapy has strong effects on R/R B-ALL patients in various disease states and is a promising immunotherapy (9, 23, 24). More and more R/R B-ALL patients are choosing CAR-T therapy for treatment. However, CAR-T therapy also has certain side effects, among which CRS is the most common and potentially life-threatening side effect (25). Consistent with previous conclusion, the determining factor that influenced the severity of CRS was tumor burden (26). National Comprehensive Cancer Network (NCCN) guidelines recommend the use of tocilizumab for the prevention and treatment of CRS caused by CAR-T cells (15). Tocilizumab is an antagonist of IL-6 receptors, which inhibits the IL-6 pathway by binding to IL-6R, thereby inhibiting inflammation (27). There are many researches on using tocilizumab for the prevention and treatment of CAR-T related CRS in patients with myeloma and lymphoma (16–18). B-ALL patients may have fewer reports due to their complex disease conditions, and due to the price, it is more likely to use tocilizumab for therapeutic rather than prevention of CRS in actual clinical practice. And the side effects of using tocilizumab to treat CRS have not yet been determined.

Our study enrolled 45 R/R B-ALL patients who received CD19 CAR-T cell therapy. We divided the patients into two cohorts based on whether or not they have received tocilizumab, with 17 patients receiving tocilizumab treatment for CRS. According to relevant guidelines and our center’s experience, tocilizumab is mainly administered within 12 hours after the onset of CRS-related symptoms (15). If there is no significant improvement after 8 hours of infusion, we will give patients a second dose. We compared baseline characteristics based on age, gender, high-risk phenotype or genotypes, lines of therapy, previous allogeneic hematopoietic stem cell transplantation (allo-HSCT), tumor burden, doses of CAR-T cells, and grade of CRS. Among them, tumor burden and the grade of CRS were higher in tocilizumab infusion cohort.

We evaluate the use of tocilizumab based on clinical symptoms such as high fever, hypoxemia, hypotension, and apply the ASTCT grading system to grade patients’ CRS. Moreover, we evaluated the efficacy of CAR-T cells on day 28 and long-term survival. In this trial, there was no significant difference in efficacy and long-term survival between 2 groups. This is consistent with the conclusions of several studies on the prevention and treatment of CAR-T related CRS by using tocilizumab (28, 29).

In terms of CRS duration, we compared patients with different grades of CRS separately. There was no statistically significant difference in the duration of CRS between toci cohort patients and non-toci cohort patients in grade 2. However, the use of tocilizumab in grade 3 can shorten the duration of CRS. Moreover, from the statistical perspective, using tocilizumab to treat CRS did not affect the expansion of CAR-T cells in patient’s body, although it may be associated with the peak expansion of CAR-T cells (30).

Regarding the potential side effects of using tocilizumab for treatment, our study shows that there is no significant difference between the two groups of patients (toci group and non-toci group) in terms of severe infection, hematological toxicity, liver function damage, and kidney function damage. Previous clinical trials have shown that tocilizumab may lead to neutropenia in patients (31), but researchers do not consider it to be a form of myelosuppression (32). Our study also considers that the infusion of tocilizumab for the treatment of CRS in B-ALL patients after CAR-T therapy has no hematological toxicity. Besides, due to the blockade of IL-6 receptors by tocilizumab, peripheral blood IL-6 levels may increase. Some studies also suggest that it may cause damage to the blood-brain barrier, allowing inflammatory factors and CAR-T cells to enter the central nervous system, thereby potentially increasing the incidence of ICANS (27, 33–35). Although, in our study, no patients experienced ICANS due to a significant increase in peripheral blood IL-6 levels by using tocilizumab. In these associations, if there is a sharply increase in IL-6 levels or no relief in CRS symptoms after the using of tocilizumab, our team will use corticosteroid as early as possible for these patients to avoid life-threatening adverse events. Some guidelines also have similar views: for severe CRS patients, it is recommended to use dexamethasone 10mg/6h for 1-3 days (20mg/6h for 3-7 days in severe cases) after treatment with tocilizumab (8mg/kg, 800mg max) is ineffective; the preferred drug treatment for ICANS is dexamethasone or methylprednisolone (15). In our center, for patients with severe ICANS after CAR-T therapy, we also used intrathecal injection of dexamethasone, which achieved excellent therapeutic effects. Corticosteroids have a wider range of immunosuppressive effects than tocilizumab, and the risk of reducing CAR-T cell expansion and efficacy may be higher [although many studies have shown that the use of corticosteroid did not affect the progression-free survival of infusion patients (36, 37)]. Meanwhile, the CAR-T expansion of few patients in our study was affected after corticosteroid infusion (**Supplementary Figure S3**), which may be related to prior treatment regimens. This conclusion is consistent with the findings of several other studies indicating that corticosteroids do not influence the efficacy of CAR-T cells (28, 38, 39). However, when we choose corticosteroid therapy, attention should be paid to the administration time and duration (40–42). We still consider tocilizumab infusion as the first treatment for CRS.

The limitation of our research is that it was a single-center retrospective cohort design with a small number of participants and multiple interfering factors (eg., the infusion time of tocilizumab is mostly based on physician experience within 12 hours after symptom appearance).

## Conclusion

In summary, despite some limitations, we have confirmed the efficacy and safety of early use of tocilizumab to treat CRS caused by CD19 CAR-T cells in B-ALL patients. CAR-T cells are a highly efficient weapon for treating hematological malignancies, and CRS is one of its unavoidable side effects. Timely treatment after the appearance of CRS related symptoms (such as fever, hypotension, hypoxemia, etc.) is necessary to benefit patients with hematological malignancies and prolong their survival time.

## Data Availability

The raw data supporting the conclusions of this article will be made available by the authors, without undue reservation.
