# Supplementary figures and images for: Efficacy and safety of tocilizumab in managing cytokine release syndrome after CD19 CAR-T therapy for relapsed or refractory B-cell acute lymphoblastic leukemia
Source: Front Immunol. 2025 Mar 14;16:1530623. doi: 10.3389/fimmu.2025.1530623 (PMC11949925; doi:10.3389/fimmu.2025.1530623)

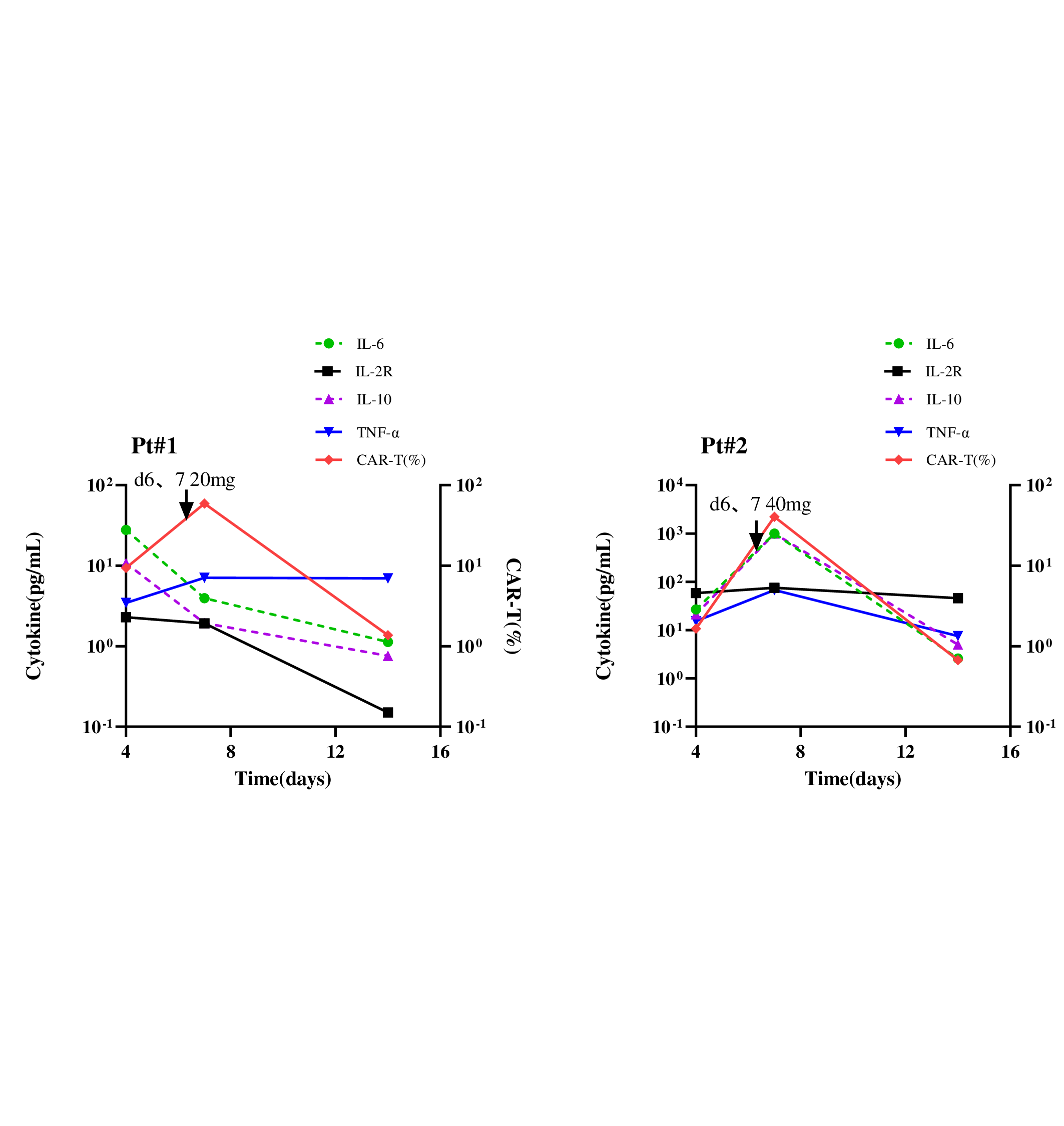

Supplement: Supplementary file 1 [file Image1.tiff]

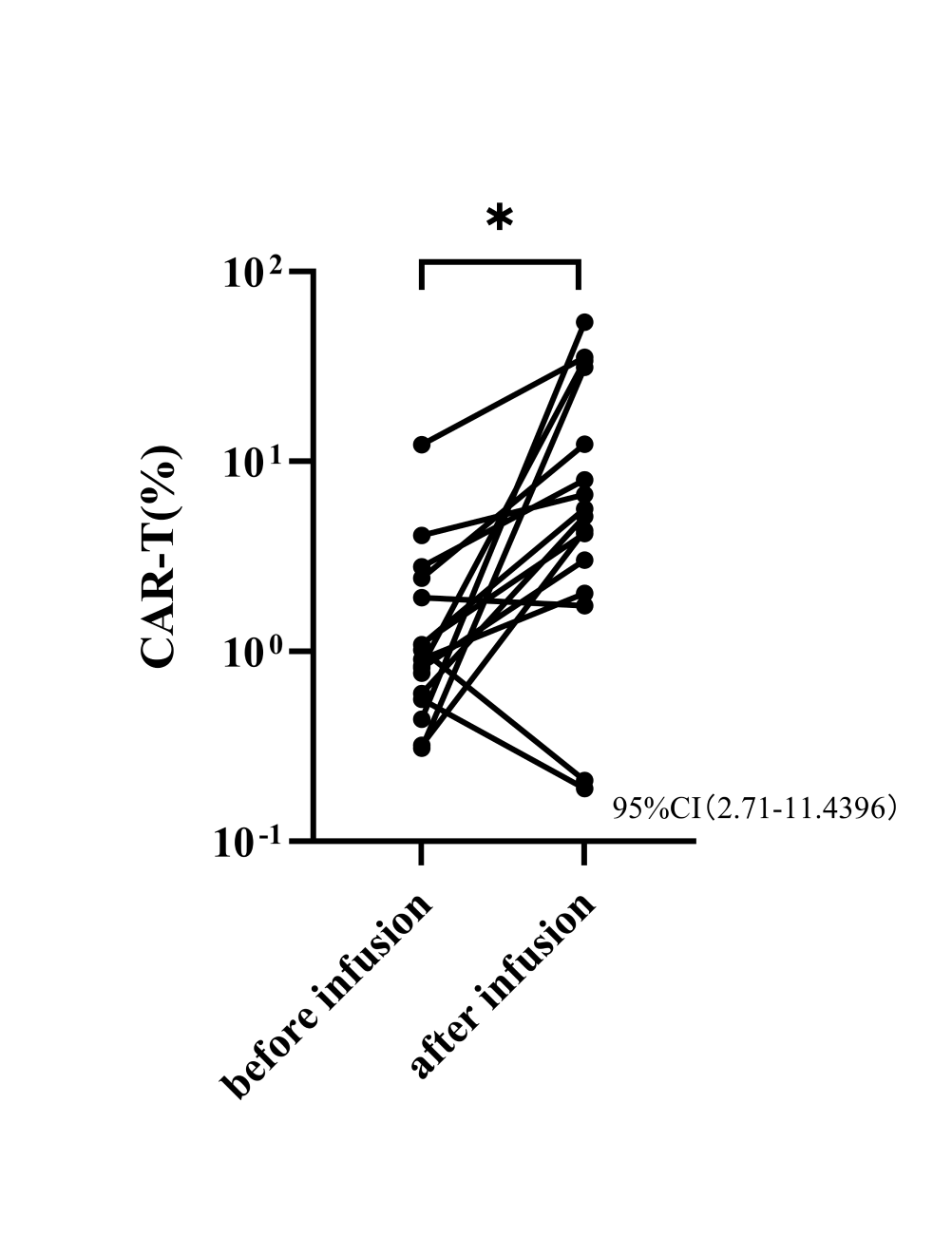

Supplement: Supplementary file 2 [file Image2.tiff]

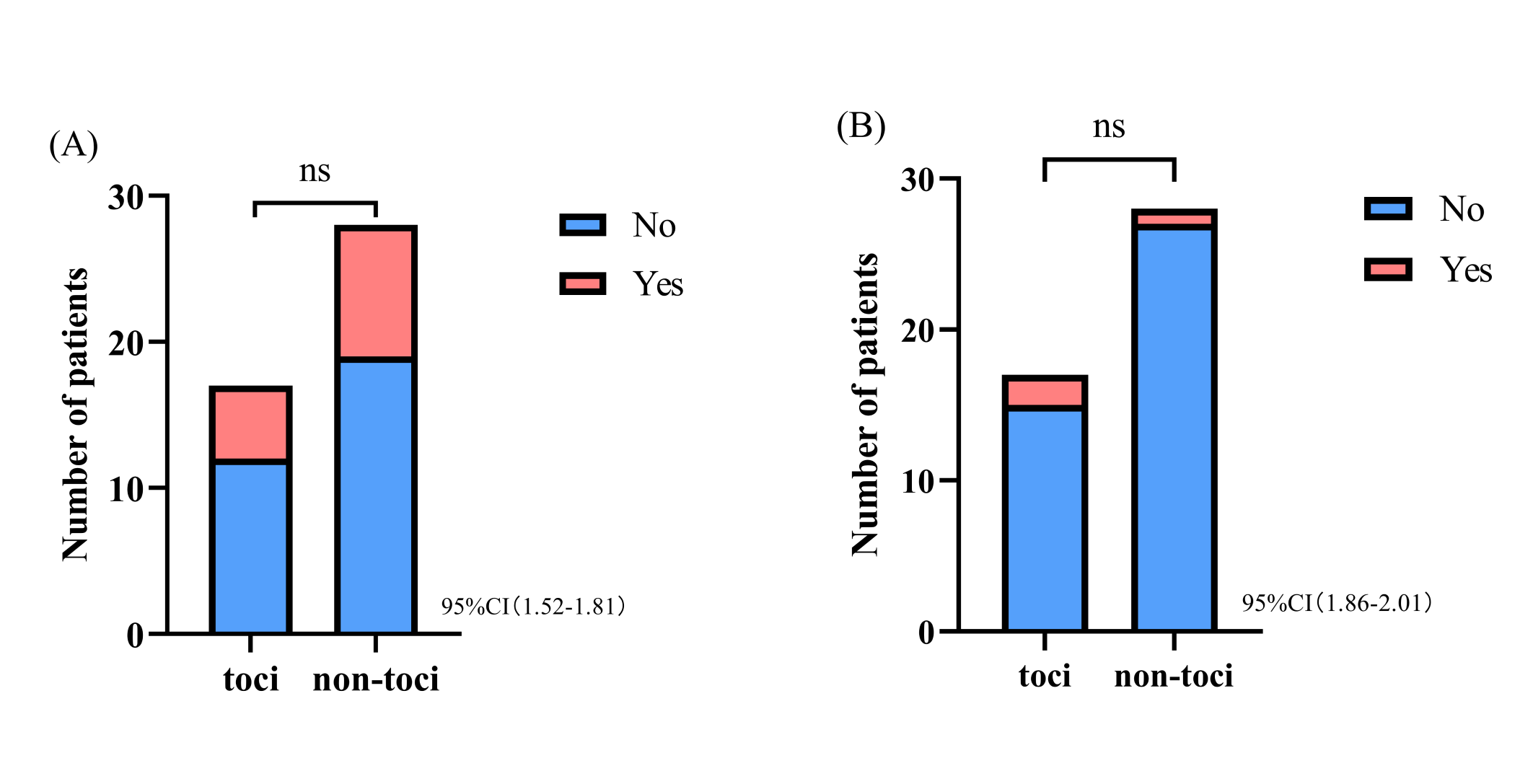

Supplement: Supplementary file 3 [file Image3.tiff]
