# Supplementary material for: Efficacy and safety of tocilizumab in managing cytokine release syndrome after CD19 CAR-T therapy for relapsed or refractory B-cell acute lymphoblastic leukemia
Source: Front Immunol. 2025 Mar 14;16:1530623. doi: 10.3389/fimmu.2025.1530623 (PMC11949925; doi:10.3389/fimmu.2025.1530623)
Supplement: Supplementary file 4 [file Table1.docx]

| Goodness of Fit | | |
| --- | --- | --- |
| -2 log likelihood | Cox & Snell R^2 | Nagelkerke R^2 |
| 34.944 | 0.423 | 0.576 |

| Hosmer-Lemeshow test | |
| --- | --- |
| χ ² | P |
| 5.067 | 0.652 |

| CHARACTERISTIC | **β** | SE | Wald | P | Exp(B) | Exp(B)95%CI | |
| --- | --- | --- | --- | --- | --- | --- | --- |
| age | 0.043 | 0.038 | 1.290 | 0.256 | 1.044 | 0.969 | 1.124 |
| sex | 0.178 | 0.985 | 0.033 | 0.856 | 1.195 | 0.173 | 8.242 |
| Grade of CRS | / | / | 0.744 | 0.863 | / | / | / |
| 1 | -19.399 | 19940.674 | 0.000 | 0.999 | 0.000 | 0.000 | / |
| 2 | -19.402 | 12212.929 | 0.000 | 0.999 | 0.000 | 0.000 | / |
| 3 | 0.957 | 1.110 | 0.744 | 0.389 | 2.605 | 0.296 | 22.962 |
| Bone marrow blasts | 0.017 | 0.015 | 1.316 | 0.251 | 1.017 | 0.988 | 1.047 |
| Median lines of therapy | -0.442 | 0.439 | 1.013 | 0.314 | 0.643 | 0.272 | 1.520 |
| High-risk phenotype or genotypes | -0.604 | 1.185 | 0.260 | 0.610 | 0.546 | 0.054 | 5.576 |
| Dose of methylprednisolone-equivalent corticosteroid | 0.008 | 0.006 | 1.855 | 0.173 | 1.008 | 0.997 | 1.019 |
