# Supplementary material for: Efficacy and safety of tocilizumab in managing cytokine release syndrome after CD19 CAR-T therapy for relapsed or refractory B-cell acute lymphoblastic leukemia
Source: Front Immunol. 2025 Mar 14;16:1530623. doi: 10.3389/fimmu.2025.1530623 (PMC11949925; doi:10.3389/fimmu.2025.1530623)
Supplement: Supplementary file 5 [file Table2.doc]

Supplementary Materials

**S1 Fig.** CAR-T ratio before and after infusion of tocilizumab. There is a statistical difference, as the proportion of CAR-T cells before infusion is lower than that after infusion, which may be related to the peak expansion of CAR-T cells in vivo.

**S2 Fig.** Liver and kidney function of two groups. There is no significantly difference between two groups.

**S3 Fig.** Cytokine, ferritin, CRP, CAR-T ratio of 2 patients using corticosteroids to treat CRS In these two patients, corticosteroids were used on days 6 and 7 respectively, and the use of corticosteroids had a significant impact on CAR-T expansion.

**S4 Table.** The logistic regression analysis of clinical characteristics in the toci group and non-toci group.
